# Individual differences in response to uncertainty and decision making: The role of behavioral inhibition system and need for closure

**DOI:** 10.1007/s11031-015-9478-x

**Published:** 2015-03-10

**Authors:** Katarzyna Jaśko, Aneta Czernatowicz-Kukuczka, Małgorzata Kossowska, Anna Z. Czarna

**Affiliations:** Institute of Psychology, Jagiellonian University, al. Mickiewicza 3, 31-120 Kraków, Poland

**Keywords:** Behavioral inhibition system, Need for cognitive closure, Decision making

## Abstract

In two studies, we examined the influence of behavioral inhibition system (BIS) and need for closure (NFC) on information processing in decision making. We expected that BIS would regulate behavior in a decisional context and that this relationship would be mediated by epistemic motivation expressed by NFC. In addition, drawing on contradictory findings in the literature on anxiety, NFC, and information processing, we investigated the moderating role of decision rules. The results supported our predictions. BIS was strongly and positively related to NFC, and through NFC it was related to decision-making style. Moreover, decision task characteristics moderated the relationship between NFC and decision making. When a task did not offer a confident decision rule, high NFC participants prolonged the information search more than low NFC individuals. However, when a reliable strategy was suggested, high NFC participants behaved in line with it. These results are discussed within an uncertainty management framework.

## Introduction

Decision making involves a considerable amount of uncertainty. When faced with a choice, a person may not know all of the available options, may be unable to recognize the quality of each option, or may select an ineffective decision making strategy, all of which create stressful and psychologically demanding conditions. One variable that is responsible for the regulation of behavior in such situations is the behavioral inhibition system (BIS; Gray and McNaughton 2003). Another variable worth studying in this context is a person’s need for closure, which expresses a motivated tendency to form clear and unambiguous judgments (NFC, Webster and Kruglanski 1994). In this paper, we examined whether individual differences in self-reported BIS, due to its relevance for self-regulation in uncertain and ambiguous situations, predict decision-making style and whether this effect is mediated by NFC. Moreover, as previous studies on the effects of anxiety and NFC on judgment and decision making offer contradictory findings, we investigated the moderating role of task characteristics on information processing during decision making.

### Individual differences in sensitivity to uncertainty and decision making

Past research has confirmed that feelings of uncertainty are related to increased physiological stress markers (Greco and Roger 2003) and cardiovascular patterns that are characteristic of response to threat (Mendes et al. 2007). One variable that is relevant to the regulation of behavior in uncertain situations is the behavioral inhibition system (BIS), which controls reactions to conflicting, ambiguous or novel stimuli and is responsible for the anxiety experienced in such situations (Gray and McNaughton 2003). The activity of BIS inhibits ongoing behavior and increases arousal and attention, which helps to determine which of incompatible or novel goals will dominate the course of action (McNaughton and Corr 2004).

Neuropsychological evidence has directly linked the experience of uncertainty with BIS. For example, BIS is related to the activity of the anterior cingulate cortex (Amodio et al. 2008), which serves to detect potential threats and expectation violations (Aston-Jones and Cohen 2005). Another study demonstrated an association between BIS and right-posterior dorsolateral prefrontal cortical activity, which is sensitive to uncertainty and ambiguity (Shackman et al. 2009). A study by Herry et al. (2007) showed that unpredictability alone was sufficient to induce neural activity in the amygdala, which is related to BIS, in both mice and humans. On a behavioral level, unpredictability elicited anxious behaviors such as avoidance and enhanced attention toward negative stimuli (Herry et al. 2007). Other studies have demonstrated a relationship between individual differences in anxiety and reactions to uncertainty (Hirsh and Inzlicht 2008; Knyazev et al. 2005). These results show that uncertainty is an aversive and stressful experience, which is positively related to anxiety controlled by BIS. As decision making involves evaluation of conflicting, risky or novel choices and is inherently associated with uncertainty, BIS may play a role in this process.

However, it is not clear which information processing style is best suited to reduce the uncertainty and anxiety experienced during decision making. Several studies have demonstrated that under conditions of uncertainty, stress and anxiety, individuals decrease information processing, form quick judgments and make simple decisions (Tversky and Kahneman 1974). For example, individuals with high trait anxiety gathered less information before making a decision and made faster decisions than individuals with low trait anxiety (Bensi and Giusberti 2007). Similarly, in an experimentally induced stress condition, subjects had a stronger tendency to end the task more quickly, engage in non-systematic scanning of information and made decisions without considering all available options compared with subjects in no stress condition (Keinan 1987). Participants under stress were also more selective when performing multi-attribute choice tasks in comparison to less aroused participants (Kossowska and Wichary 2006). Results of other studies have shown that coping with uncertainty consumes self-regulatory resources, and when uncertainty is induced people choose easier and less demanding options than when they feel certain (Alquist 2010; Milkman 2012). However, other studies have shown that uncertainty and related anxiety are associated with complex judgments and systematic decision making. For example, studies on causal uncertainty have demonstrated that people who chronically feel uncertain process information more systematically than people who chronically feel certain (Weary et al. 2001; Weary and Jacobson 1997). Tiedens and Linton (2001) demonstrated that participants were less likely to use heuristic processing when induced to feel emotions associated with uncertainty than when induced to feel emotions associated with certainty. In some cases, uncertainty can also cause people to postpone decisions until the uncertainty is resolved, which shows that quick judgment is not a preferred option when feeling uncertain (Shafir 1994). Drawing on these contradictory findings, in the present paper, we investigated whether decision task characteristics may act as possible moderators of decision making style when BIS is activated.

### Motivation to reduce uncertainty and decision making

Previously, we proposed that BIS is a system that is responsible for sensitivity to uncertainty that should affect decision making. Moreover, we believe that motivation to exit uncertain and ambiguous situations by forming quick and clear-cut judgments, i.e., NFC (Webster and Kruglanski 1994), may mediate the effect of BIS on decision making. Individuals with high NFC are intolerant of confusion and uncertainty and therefore are inclined to make rapid decisions (Kruglanski 2004). On the other hand, low NFC individuals are motivated to analyze situations in a systematic manner, to consider alternative options, and to make complex decisions (Kruglanski 2004). It could be expected that high NFC helps to shorten anxiety experienced in uncertain situations and therefore should be more pronounced when BIS is situationally activated or among people chronically high in BIS. Although this is a convincing possibility, there has been little research reported on the link between NFC and BIS. Results of one clinical study support the positive link between anxiety and NFC (Colbert et al. 2006). In addition, Roets and van Hiel (2008) found that, when unable to reach closure in a decision task, high NFC individuals expressed physiological stress such as increased heart rate, higher systolic blood pressure and increased electro-skin conductance. Results of one recent study (Corr et al. 2013) demonstrated a strong and positive link between BIS and NFC. However, this study was concentrated on social attitudes and did not examine behavioral correlates of both variables. Thus, the purpose of our paper is to examine the relationship between BIS and NFC, and to verify if NFC mediates the effect of BIS on decision making style.

Although most previous research has demonstrated that NFC is related to a simplified cognitive process involved in decision making, a limited information search, and a preference for clear and unambiguous judgment, some findings contradict this conclusion. Among the studies that demonstrate the former, Choi et al. (2008) showed that, before making a consumer choice, high NFC individuals considered less information and preferred a more simplistic and non-compensatory mode of decision making than low NFC subjects. Similar results were obtained by Webster et al. (1996), who demonstrated that participants with elevated NFC demanded less information before forming a judgment. Moreover, these individuals were more susceptible to the primacy effect than subjects with low NFC. Stronger reliance on early and accessible information under heightened NFC was also shown in studies by Kruglanski and Freund (1983). They found that both the primacy effect and anchoring were stronger under time pressure and decreased when participants expected that their judgments would be evaluated. Individuals with high NFC are also more confident in their decisions (Mayseless and Kruglanski 1987), prefer more familiar options, and experience stronger regret after choosing an unfamiliar option than individuals with low NFC (Mannetti et al. 2007).

However, there is also evidence that under specific conditions, high NFC individuals search for more information and postpone their decision to a larger extent than low NFC individuals. For example, Vermeir et al. (2002) asked participants to choose between brands of unfamiliar products to eliminate reliance on prior knowledge. In this situation, high NFC individuals searched for more information about products than low NFC individuals. Moreover, high NFC participants sought significantly more information before their opinion was crystallized than after that point, while this pattern was not found for low NFC participants. Similar findings were obtained by Houghton and Grewal (2000), who found that high NFC resulted in a less extensive information search only when the products involved in the decision were important to the participants; therefore, participants presumably had well-formed and accessible opinions. Otherwise, no differences were found between high and low NFC individuals. In another study, when the choice was easy and therefore the subjects’ confidence in their initial hypothesis was relatively high, subjects high in NFC tended to search for less information than subjects low in NFC (Kruglanski et al. 1991). However, when the choice was difficult and their initial confidence was low, subjects high in NFC searched for more information than subjects low in NFC. Similarly, Kossowska and Bar-Tal (2013) demonstrated that NFC was strongly associated with quick and simple decision-making only among individuals who expected to be able to satisfy their epistemic need, i.e., had high ability to achieve closure. For participants low in ability to achieve closure, NFC was associated with systematic processing, which was manifested by more complex and time-consuming decision making.

These studies suggest that without a satisfactory basis for closure, whether resulting from familiarity with a subject or sufficiently strong confidence in an initial guess, NFC is not in fact related to a limited information search. Only when high NFC individuals had some prior knowledge on which to base their judgment did they make a decision earlier than low NFC individuals. However, without a basis for a clear-cut judgment, high NFC individuals prolonged the searching phase to a greater extent than low NFC individuals. This finding would further suggest that the need for cognitive closure is not satisfied by any rapid and impulsive answer; rather, a particular type of answer is sought by high NFC individuals. Specifically, high NFC individuals seek the answer or decision that allows them to reduce uncertainty in a legitimate and satisfactory way. Otherwise, they should prolong their information search.

### Overview

In two studies, we tested a model of the relationship between self-reported BIS, NFC and decision making behavior. Because BIS is activated in uncertain situations, we expected that it should be related to the decision making process. We also predicted that more sensitive BIS should be associated with high NFC. Moreover, we hypothesized that the relationship between BIS and decision making style would be mediated by high NFC. In addition, drawing on the conflicting results from previous research, we explored the moderating role of task characteristics in the relationship between decision making and both BIS and NFC. We examined these relationships using an abstract and unfamiliar decision task, and we either did not inform participants about the best decision strategy or we directly manipulated the basis for closure by specifying the decision strategy. We expected that without sufficient confidence in a rule, participants high in NFC would engage in a more extensive information search before making a decision than participants low in NFC (Study 1). In contrast, when a specific decision rule was offered, we expected that an anchoring effect would be found such that participants high in NFC would behave more in line with the suggested rule than participants low in NFC (Study 2).

## Study 1

The aim of the Study 1 was to explore the relationship between self-reported BIS, NFC and behavior in an abstract and unfamiliar decision making task that offered no prior information about the best strategy by which to make decisions. We used the Information Sampling Task (Clark et al. 2006), in which participants gathered information and made a decision about the proportion of colored boxes on a board of 25 boxes. Because the task allowed participants to reduce uncertainty by gathering more information without any costs and in a short amount of time, we expected that in such conditions BIS and NFC would be related to a longer decision time and a more extensive information search. Secondly, we hypothesized that BIS would be related to a higher level of NFC and that NFC would mediate the effects of BIS on the decision-making process.

### Method

#### Participants

The study was conducted on-line. Participants were 115 students from a large Polish university recruited via a student mailing list (97 women, 17 men, 1 person did not report gender, *M*age = 21.42, *SD* = 3.89). In exchange for their participation, each participant could win one of four tickets in a lottery (each worth approximately $15).

#### Materials and procedure

The study was programmed in Inquisit software (Inquisit 2013). Participants completed BIS and NFC scales and then performed the Information Sampling Task (Clark et al. 2006).

##### Behavioral inhibition system

BIS was measured with Carver and White’s 20-item BIS/BAS questionnaire (Carver and White 1994; Polish translation: Müller and Wytykowska 2005). In subsequent analyses, we only used the BIS subscale, which consists of seven items (α = 0.72). A sample item is “I worry about making mistakes.” Participants indicated how much they agreed with each item on a scale from 1 (strongly agree) to 4 (strongly disagree). The items were reversed such that higher scores indicated higher BIS.

##### Need for cognitive closure

NFC was measured with the 27 items of Webster and Kruglanski’s scale (1994; Polish translation: Kossowska 2003). The scale includes four subscales: Preference for Order, Preference for Predictability, Discomfort with Ambiguity, and Closed-mindedness. The fifth subscale, Decisiveness, was replaced with six items developed by Roets and van Hiel (2007) because the original subscale has been recognized as measuring ability to achieve cognitive closure instead of motivation. A sample item is “I don’t like situations that are uncertain.” Participants indicated their responses on a scale from 1 (completely disagree) to 6 (completely agree). The Closed-mindedness subscale was excluded due to its low reliability, and the overall index was calculated using only four other subscales (α = 0.85).

##### Information sampling task (IST)

To measure the decision making process, we used a task developed by Clark et al. (2006). Participants were presented with a 5 × 5 matrix of 25 grey boxes and two colored panels at the foot of the screen (e.g., yellow and blue). When a participant clicked on a grey box, it revealed one of two colors and remained open for the entire trial. The task was to decide which of the two colors prevailed on the board. Participants could open as many boxes as they wanted before making their decision. They won points for each correct decision and lost points for an incorrect decision. Once they decided which color was in the majority, participants clicked the panel with this color. At that point, the remaining boxes were uncovered, and participants received feedback about points earned or lost.

The task consisted of two rounds presented in a counterbalanced order with ten trials in each round. In the Fixed Wins (FW) round, participants could win 100 points if they made a correct decision, irrespective of the number of opened boxes, and they lost 100 points if they were wrong. In the Decreasing Wins (DW) round, there were costs of opening boxes such that the maximum amount to win (250 points) decreased by ten points for every box opened. Again, after an incorrect decision, participants lost 100 points, regardless of the number of boxes opened. A test trial took place before each round. Although we expected that effects would occur in the FW round when there were no costs of gathering information, we included the DW condition to explore the effects of NFC under conditions of trade-off between uncertainty and loss. Decision behavior was measured by the average number of boxes opened, the decision time, and the number of correct judgments made in each condition. The total points won indicated overall task performance. In the present article, we will focus on information search as indicated by the number of boxes opened and decision time. Decision time was measured from the moment participants were first presented with the matrix to the moment they made a decision. It was log transformed.

### Results

To verify whether BIS and NFC were related to information search and decision time we analyzed the pattern of correlations between those variables. In the second step we tested the mediation model for each of the dependent variables. Both analyses were conducted separately for the FW condition and the DW condition. Table 1 presents descriptive statistics and correlations between variables.

**Table 1:** Intercorrelations, means, and standard deviations (Study 1)

	BIS	NFC	Decision time (FW^a^)	Decision time (DW^a^)	Opened boxes (FW)	Opened boxes (DW)
BIS						
NFC	0.49^***,b^					
Decision time (FW)	0.07	0.20^*^				
Decision time (DW)	0.09	0.19^*^	0.58^***^			
Opened boxes (FW)	0.05^b^	0.16^b^	0.62^***^	0.31^***^		
Opened boxes (DW)	0.05	0.14	0.40^***^	0.74^***^	0.46^***^	
*Mean*	3.12	3.79	3.97	3.89	16.69	9.60
*SD*	0.53	0.52	0.22	0.26	5.64	4.69
Range	1.86–4.00	2.37–5.00	3.50–4.52	3.34–4.68	3.80–25.00	0–20.80
Possible range	1–4	1–6	–	–	0–25	0–25

*N* = 115

* *p* < 0.05; ^***^ *p* < 0.001

^a^FW refers to the Fixed Wins condition and DW refers to the Decreasing Wins condition

^b^Due to the exclusion of an outlier in the model of relationships between BIS, NFC, and the number of opened boxes (*n* = 114) the correlations for this model are different: between BIS and NFC (*r* = 0.51, *p* < 0.001), NFC and Opened boxes (FW) (*r* = 0.20, *p* = 0.03), BIS and Opened boxes (FW) (*r* = 0.03, *p* = 0.757)

#### Number of opened boxes

**Fig. 1:**
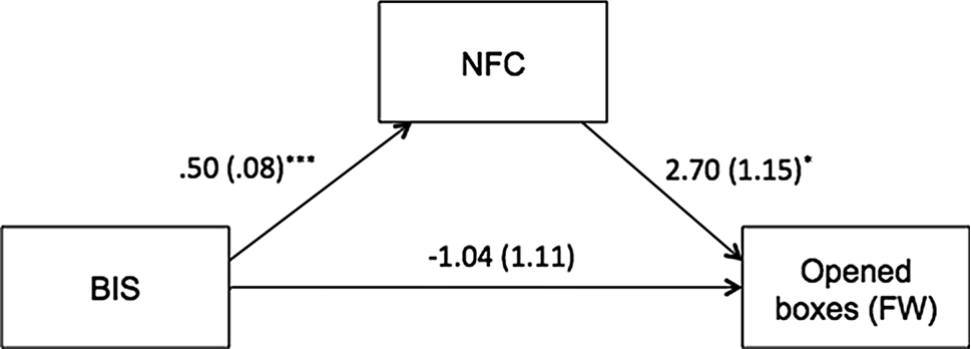
Mediation model of the relationship between BIS, NFC, and number of opened boxes (Fixed Wins condition) in Study 1 (^*^ *p* < 0.05, ^***^ *p* < 0.001)

In both FW and DW rounds bivariate correlations between BIS and the number of opened boxes and NFC and the number of opened boxes were not significant. To test the simultaneous effects of BIS and NFC on information search, we used the PROCESS program (Hayes 2013). We tested mediation models separately for the FW and DW rounds. We controlled for the order of rounds, but it was not significant; thus, this variable was dropped from the analysis. In the analysis for the FW round we excluded one case that had a much stronger influence on the model parameters than other cases (as indicated by Cook’s distance, studentized residuals, and DFFIT).^1^ In line with our hypothesis, BIS had a significant and positive effect on NFC (*b* = 0.50, *SE* = 0.08, *β* = 0.51, *p* < 0.001). NFC had a positive effect on the number of opened boxes (*b* = 2.70, *SE* = 1.15, *β* = 0.25, *p* = 0.02). Neither the direct effect of BIS on the number of opened boxes (*b* = −1.04, *SE* = 1.12, *β* = −0.10, *p* = 0.357), nor the total effect (*b* = 0.31, *SE* = 0.98, *β* = 0.03, *p* = 0.757) were significant. The entire model was marginally significant [*F*(2, 111) = 2.83, *p* = 0.063, *R* ^2^ = 0.05]. More importantly, the indirect effect of BIS on the number of opened boxes estimated with 20,000 bootstrapped samples was significant [*b* = 1.34, 95 % CI (0.23, 2.83)], which indicates that BIS was related to information search through its impact on NFC. The mediation model is presented in Fig. 1. There were no significant effects of NFC and BIS on the number of opened boxes in the DW round.

#### Decision time

Correlations between BIS and decision time were not significant (in the FW round: *r* = 0.07, *p* = 0.451; in the DW round: *r* = 0.09, *p* = 0.363) but correlations between NFC and decision time were significant in both rounds (FW: *r* = 0.20, *p* = 0.029; DW: *r* = 0.19, *p* = 0.045).

In the mediational analysis for the FW condition BIS had a significant and positive effect on NFC (*b* = 0.48, *SE* = 0.08, *β* = 0.48, *p* < 0.001). NFC had a positive effect on decision time (*b* = 0.09, *SE* = 0.04, *β* = 0.22, *p* = 0.038). Neither the direct effect of BIS on decision time (*b* = −0.02, *SE* = 0.04, *β* = −0.04, *p* = 0.733) nor the total effect (*b* = 0.03, *SE* = 0.04, *β* = 0.07, *p* = 0.451) were significant. The entire model was marginally significant [*F*(2, 112) = 2.48, *p* = 0.088, *R* ^2^ = 0.04]. Although the confidence intervals for the indirect effect estimated with 20,000 bootstrapped samples included zero [*b* = 0.04, 95 % CI (−0.001, 0.10)], the Sobel test for this indirect effect was marginally significant (*b* = 0.04, *SE* = 0.02, *p* = 0.052). The mediation model for the DW round was not significant (the Sobel test *b* = 0.04, *SE* = 0.03, *p* = 0.089).

### Discussion

The results obtained in the first study demonstrated atypical effects of NFC on the decision-making process. Despite the fact that in previous studies NFC was usually related to simplified information processing (Choi et al. 2008; Kruglanski and Freund 1983), in our study in the round without costs of information (FW) high NFC individuals engaged in an information search to a greater extent than low NFC individuals. We think that high NFC participants opened more boxes and spent more time on decision making, because with relatively little effort and no costs they could greatly reduce uncertainty regarding the correct decision. Even opening all boxes on a matrix, which in this task allowed participants to achieve absolute certainty, did not take much longer than a more limited information search.

We believe that this effect is due to the specificity of the decision task that offered a single rule—opening more boxes—on which high NFC individuals could base their decision. This effect is in line with previous studies that showed higher cognitive engagement among high NFC individuals when there was no prior knowledge that would reduce their uncertainty (Vermeir et al. 2002). However, although this is an intuitively appealing explanation, it needs to be tested directly. This was the goal of the Study 2, in which we manipulated the presence/absence of a decision rule. Moreover, despite the fact that direct effects of NFC on information search and decision time were significant, the models were only marginally significant. Thus, the aim of the Study 2 was to replicate the basic effect of NFC on decision making.

The effect of NFC on the information search was significant in the FW round where no costs were associated with information search, but it was weaker and not significant in the DW round when information was costly. This difference might suggest that high NFC individuals engage in a more extensive information search when no additional trade-off is necessitated between reducing uncertainty and increasing gains. However, such conclusions must be treated with some caution, as the observed effects in both conditions were rather weak. Thus, we decided to keep both conditions in Study 2 in order to replicate the effects and further investigate the possible difference between them.

In line with our expectations, we observed a strong and positive relationship between BIS and NFC. This finding suggests that NFC may be one of the motivational mechanisms by which individuals who are sensitive to uncertainty regulate their interactions with a stressful environment. NFC as a motivation to form clear-cut and unambiguous judgments may decrease anxiety experienced in such situations by individuals with heightened BIS activity.

We found a significant indirect effect of BIS through NFC on the information search. However, with regard to decision time, this effect was not significant. We believe that those results offer partial support to the hypothesis of the effects of uncertainty on decision making in terms of heightened NFC. In Study 2, we aimed to replicate those effects and directly test their boundary conditions. Contrary to our hypotheses, the total effect of BIS on our dependent variables was not significant. It could result from the fact that the indirect effect of BIS on decision making process was positive but the direct effect of BIS, though insignificant, was in the opposite direction.

## Study 2

The goals of Study 2 were to replicate the results of Study 1 and to verify whether the relationship between BIS, NFC, and information search would change depending on the presence or absence of an explicit decision rule. To achieve these goals, we manipulated the information given to participants about the appropriate strategy to use in the decision-making task. We expected that participants high in BIS and NFC would behave more in line with the suggested decision rule than participants low in BIS and NFC because information that decreases the level of uncertainty is more important for the former individuals. Specifically, we hypothesized that in comparison to individuals low in BIS and NFC those high in BIS and NFC would prolong their searching process and would inspect more information when offered a rule that encouraged them to search for more information. However, when told that the best rule is to limit the information search, they would decrease the amount of acquired information and shorten their decision time. In the control condition, when no information regarding the optimal way of solving the task was presented to participants, we aimed to replicate the results obtained in Study 1. Moreover, we expected that the effect of BIS on decision time and information search would be mediated by NFC. We further explored the effect of rounds differing in costs incurred for information search (Fixed Wins vs. Decreasing Wins) to parallel Study 1.

### Method

#### Participants

The study was conducted on-line. Two hundred twenty-three participants were recruited via invitation placed on a popular free ads web portal (185 women, 38 men, *M*age = 23.30, *SD* = 5.59). In exchange for their participation, each participant could win one of eight tickets in a lottery (each worth approximately $15).

#### Materials and procedure

The study was programmed in Inquisit software (Inquisit 2013). BIS (α = 0.73) and NFC (α = 0.89) were measured with the same scales as in Study 1. The only change was introduced in the IST task, in which we manipulated the decision rule in both rounds (FW and DW).

##### Manipulation of the decision rule

We manipulated the content of the decision rule by presenting participants with information that ostensibly described how other people perform in this task. In the high anchor condition before conducting the FW round, participants read: “People who do this task on average open 24 boxes. Their accuracy level is 90 %.” Before the DW round, the number “24” was substituted with “15” to adjust it to the fact that people in the DW round opened fewer boxes on average than in the FW round. In the low anchor condition, the information stated: “People who do this task on average open eight boxes. Their accuracy level is 90 %.” Before the DW condition, “eight” was substituted with “five.” Information about accuracy was introduced to strengthen the persuasiveness of the information. In the control condition no information about average number of boxes opened was offered. We dummy-coded the manipulation variable with a control condition as a reference group, which produced two variables: Low Anchor Condition and High Anchor Condition.

##### Manipulation check

To verify whether participants remembered the information about the rule throughout the task, we asked them at the end to state how many boxes other participants opened during this task.

### Results

#### Manipulation check

A one-way ANOVA revealed a significant effect of condition on the manipulation check items [In the FW round: *F*(2, 220) = 103.11, *p* < 0.001, *η* ^2^ = 0.49; in the DW round: *F*(2, 220) = 83.80, *p* < 0.001, *η* ^2^ = 0.43]. Participants in the low anchor condition remembered that other people opened fewer boxes (FW: *M* = 7.95, *SD* = 3.83; DW: *M* = 5.47, *SD* = 2.49) than participants in high anchor condition (FW: *M* = 20.99, *SD* = 5.72; DW: *M* = 14.84, *SD* = 6.08). In the control condition, participants thought that others opened 13.58 boxes (*SD* = 6.73) in the FW round and 7.69 (*SD* = 4.55) in the DW round.

#### Number of boxes opened

In the control condition NFC was positively related to the number of opened boxes in the FW round, but the effect in the DW round was not significant. In the high anchor condition the effect was in the similar direction but was not significant and in the low anchor condition this relationship was negative and not significant. BIS was positively related to number of opened boxes only in the control condition for the FW round. Table 2 presents descriptive statistics and correlations between variables separately for the three conditions.

**Table 2:** Intercorrelations, means, and standard deviations (Study 2)

	BIS	NFC	Decision time (FW^a^)	Decision time (DW^a^)	Opened boxes (FW)	Opened boxes (DW)
High Anchor (n = 79)						
BIS						
NFC	0.43^***^					
Decision time (FW)	−0.02	0.27^*^				
Decision time (DW)	−0.02	0.22	0.75^***^			
Opened boxes (FW)	0.01	0.19	0.63^***^	0.31^*^		
Opened boxes (DW)	−0.08	0.15	0.45^***^	0.63^***^	0.54^***^	
*Mean*	3.08	4.02	3.93	3.86	14.81	10.03
*SD*	0.48	0.59	0.29	0.30	6.00	4.51
No Anchor (n = 77)						
BIS						
NFC	0.49^***^					
Decision time (FW)	0.14	0.33^***^				
Decision time (DW)	−0.01	0.12	0.67^***^			
Opened boxes (FW)	0.26^*^	0.27^*^	0.76^***^	0.44^***^		
Opened boxes (DW)	0.17	0.08	0.40^***^	0.69^***^	0.49^***^	
*Mean*	3.10	3.81	3.84	3.75	14.28	8.70
*SD*	0.52	0.70	0.28	0.29	6.57	4.33
Low Anchor (n = 78)						
BIS						
NFC	0.39^***^					
Decision time (FW)	−0.10	−0.17				
Decision time (DW)	0.03	−0.12	0.80^***^			
Opened boxes (FW)	−0.06	−0.22	0.66^***^	0.39^***^		
Opened boxes (DW)	0.11	0.00	0.52^***^	0.60^***^	0.59^***^	
*Mean*	3.09	3.85	3.76	3.69	10.81	6.88
*SD*	0.46	0.67	0.31	0.28	6.54	4.41

*  *p* < 0.05; *** *p* < 0.001

^a^FW refers to the Fixed Wins condition and DW refers to the Decreasing Wins condition

**Fig. 2:**
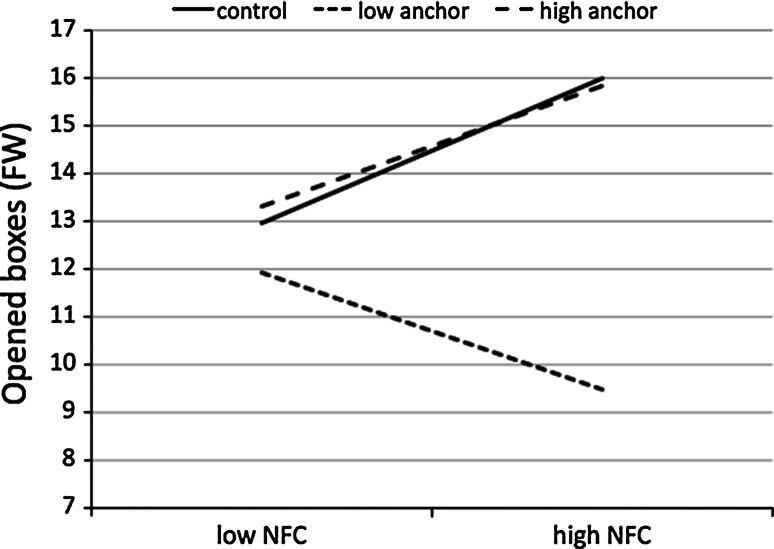
Number of opened boxes (Fixed Wins condition) as a function of NFC and rule manipulation (Study 2)

We used the PROCESS program to test the interactional effect of NFC and the manipulation, and the indirect effect of BIS on the information search through NFC (Model 16; Hayes 2013). In the FW round the model was significant [*F*(7, 215) = 6.96, *p* < 0.001, *R* ^2^ = 0.18]. In line with our expectations, we found that BIS had a positive effect on NFC (*b* = 0.58, *SE* = 0.08, *β* = 0.43, *p* < 0.001). We also found a significant interaction between NFC and the experimental manipulation (NFC × Low Anchor Condition: *b* = −4.16, *SE* = 1.48, *β* = −0.42, *p* = 0.005; NFC × High Anchor Condition: *b* = −0.38, *SE* = 1.55, *β* = −0.04, *p* = 0.80; for both interactions, *R* ^2^-change = 0.035, *F*(2, 215) = 4.60, *p* = 0.011). The effect of NFC on the number of opened boxes was positive and significant in the control condition (*b* = 2.30, *SE* = 1.06, *β* = 0.23, *p* = 0.031). The effect was positive but not significant in the high anchor condition (*b* = 1.91, *SE* = 1.22, *β* = 0.19, *p* = 0.119) and was negative but not significant in the low anchor condition (*b* = −1.86, *SE* = 1.10, *β* = −0.19, *p* = 0.094). These results are presented in Fig. 2.

The direct effect of BIS on the number of opened boxes was not significant (*b* = 0.76, *SE* = 0.93, *β* = 0.06, *p* = 0.419). However, the indirect effect estimated with 20,000 bootstrapped samples was significant in the control condition [*b* = 1.33; 95 % CI (0.23, 2.73)]. This effect was positive but not significant in the high anchor condition [*b* = 1.11, 95 % CI (−0.36, 2.77)], and it was negative but not significant in the low anchor condition [*b* = −1.07, 95 % CI (−2.50, 0.15)]. We also performed a test of moderated mediation (Hayes 2013). The difference in the indirect effects between the control condition and the high anchor condition was not significant [95 % CI (−2.09, 1.50)], but it was significant between the control condition and the low anchor condition [95 % CI (−4.34, −0.74)].

The effect of order of rounds was significant (*b* = −3.30, *SE* = 0.82, *β* = −0.50, *p* < 0.001), which means that participants opened more boxes in the FW round when this round was first. There were no significant effects of NFC and BIS on number of opened boxes in the DW condition.

#### Decision time

In the high anchor and control conditions NFC was positively related to decision time in the FW round, but those effects in the DW round were not significant. In the low anchor condition the relationship between NFC and decision time (FW and DW) was negative and not significant. However, the correlations between BIS and decision time in both rounds were not significant.

**Fig. 3:**
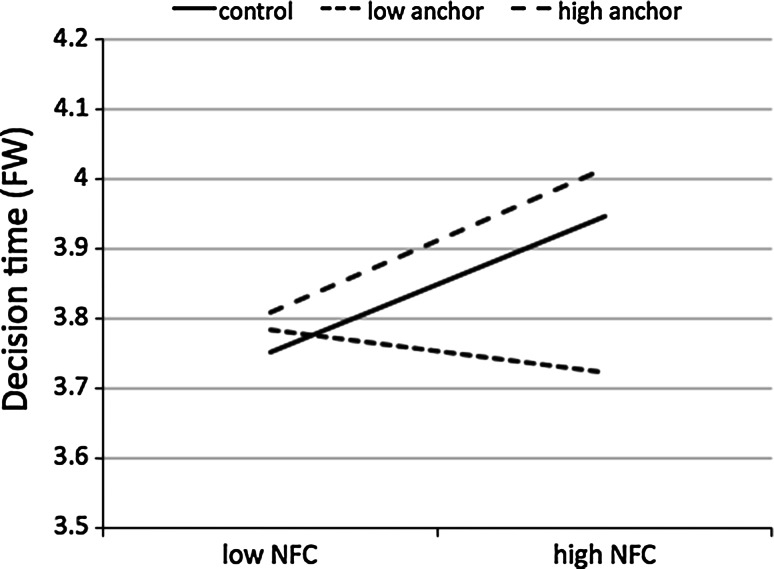
Decision time (Fixed Wins condition) as a function of NFC and rule manipulation (Study 2)

The mediation model was significant in the FW round [*F*(7, 215) = 7.10, *p* < 0.001, R^2^ = 0.19]. BIS had a positive effect on NFC (*b* = 0.58, *SE* = 0.08, *β* = 0.43, *p* < 0.001). We found a significant interaction between NFC and the experimental manipulation (NFC × Low Anchor Condition *b* = −0.19, *SE* = 0.07, *β* = −0.43, *p* = 0.004; NFC × High Anchor Condition *b* = 0.01, *SE* = 0.07, *β* = 0.02, *p* = 0.902; for both interactions, *R* ^2^-change = 0.04, *F*(2, 215) = 5.41, *p* = 0.005). Further analyses showed that the effect of NFC on decision time (in FW) was positive and significant in the control condition (*b* = 0.15, *SE* = 0.05, *β* = 0.32, *p* = 0.003) and in the high anchor condition (*b* = 0.16, *SE* = 0.06, *β* = 0.33, *p* = 0.005), but it was negative and not significant in the low anchor condition (*b* = −0.05, *SE* = 0.05, *β* = −0.10, *p* = 0.357). The visualization of this interaction is presented in Fig. 3.

Although the direct effect of BIS on decision time (FW) was not significant (*b* = −0.04, *SE* = 0.04, *β* = −0.06, *p* = 0.382), the indirect effect estimated with 20,000 bootstrapped samples was significant in the control condition [*b* = 0.08; 95 % CI (0.04, 0.15)] and in the high anchor condition [*b* = 0.09, 95 % CI (0.03, 0.18)]; however, this effect was not significant in the low anchor condition [*b* = −0.03, 95 % CI (−0.09, 0.03)]. The difference in the indirect effects between the control condition and the high anchor condition was not significant [95 % CI (−0.08, 0.09)], but it was significant between the control condition and the low anchor condition [95 % CI (−0.20, −0.04)].

Finally, the effect of order of rounds was significant (*b* = −0.15, *SE* = 0.04, *β* = −0.50, *p* < 0.001), which means that decision time in the FW round was shorter when this round was second. There were no effects of NFC and BIS on decision time in the DW condition.

### Discussion

In the control condition of Study 2, when no anchor was offered to participants, we obtained a similar pattern of results as in Study 1. In the FW round in this condition high NFC subjects gathered more information and spent more time making a decision than low NFC individuals. In the high anchor condition, we obtained the same pattern of results and this group was not significantly different from the control group. However, when an available decision rule suggested that a limited information search was the strategy successfully employed by other participants (low anchor condition), the effects of NFC with regard to decision time and number of opened boxes were not significant. These results are consistent with our expectations that high NFC individuals would behave in line with accessible and reliable rules. Our experimental manipulation induced differences among high NFC individuals but individuals low in NFC were not affected by it. It is an interesting result, because in the high anchor condition the suggested style of processing—longer decision making process and extended information search—was opposite to the one usually associated with high NFC. We think that this anchoring effect occurred because such rules decrease uncertainty, which is important for high NFC individuals. This result corroborates past research showing how psychological states similar to NFC (e.g., cognitive load, low need for cognition) resulted in stronger judgmental anchoring and insufficient adjustment (Epley and Gilovich 2006).

In Study 2, we replicated the strong and positive relationship between NFC and BIS found in Study 1. More importantly, in the FW round we also replicated the indirect effect of BIS on decision making in the control group. This pattern of results offers support for our initial assumption that the effects of sensitivity to uncertainty on decision making result from avoidance of uncertainty and a stronger motivation to reach closure among highly uncertain individuals. Significant moderation of this indirect effect by situational conditions indicates that when the available information suggested that a prolonged search was the best strategy, high BIS individuals, due to their higher level of NFC, gathered more information to reduce uncertainty than low BIS individuals. However, the rule available in the low anchor condition, which advised a limited information search, diminished the indirect relationship between BIS and a prolonged decision making strategy. We think that in this condition, reduction of uncertainty to an acceptable level was achieved with a shorter decision making process. A stronger manipulation may be needed to significantly reverse this effect.

Although no difference was found between the control group and the high anchor group, we think that there still may be some psychological differences between those two conditions. In the high anchor condition, the decision rule was externally given to participants, while in the control condition and in the Study 1 it was not available from the outside. Therefore, it is possible that the control condition and high-anchor condition may differ with regard to other aspects of the decision making process such as confidence in the decision or satisfaction with a decision, even though on a behavioral level the groups were similar. For example, it could be expected that in the high anchor condition, the uncertainty regarding the chosen strategy was lower than in the control condition. It should be noted that the abovementioned effects were restricted to FW condition only. We will discuss this limitation in the general discussion.

## General discussion

We began this article with the observation that decision making can be a demanding activity, but at the same time not everyone experiences the process in the same way. In two studies, we investigated the effects of individual differences in BIS and NFC on the extent of information gathering involved in a decision making process. In both studies, BIS was strongly and positively related to NFC. This result confirms the relationship between BIS and NFC, which was found in one recent study (Corr et al. 2013).

Moreover, BIS was related to decision making behavior through NFC. This indirect effect is in line with the interpretation of BIS as a system that controls reactions to new and ambiguous situations (Amodio et al. 2008; Shackman et al. 2009). If uncertainty evokes more negative reactions among high BIS individuals (Hirsh and Inzlicht 2008), then a high level of NFC offers a way out of such situations, either by premature closure or by extensive gathering of information, depending on the situational context. It should be noted that neither direct nor the total effect of BIS on decision making were significant. It could be that we were not able to detect such effects of BIS even though we were able to obtain a significant indirect effect, because the statistical power is higher in the test of the indirect effect than it is in the test of the total and direct effect (Kenny and Judd 2014). As the observed effects were moderated it would be interesting to investigate other boundary conditions that would strengthen the relationship between BIS, NFC, and decision making. One of such variables could be framing the decisions in terms of gains or losses. As high BIS individuals are more sensitive towards negative stimuli they could be more motivated to achieve certainty when the decision could result in loss than when it could result in a gain.

Our interpretation of the relationship between BIS activity and NFC focuses on the functionality of NFC, which we assume is one of the mechanisms applied by highly uncertain individuals to cope with a stressful environment. However, further research is needed to determine whether behavior elicited by high NFC indeed reduces feelings of uncertainty and anxiety. In addition, although the causal direction assumed in the present study seems convincing in terms of the biological underpinnings of BIS, future research should verify the causal relationship between uncertainty and NFC with an experimental design. To fully understand the nature of the relationship between BIS and decision making, further studies are definitely needed.

With regard to the relationship between NFC and decision making process, in both of our studies, when a decision task did not offer a clear and confident cue, high NFC participants sought more information and spent more time making a decision than low NFC individuals. In contrast, when a strategy was suggested, high NFC participants behaved in line with the suggested strategy. This result is important, because it confirms that NFC is not always related to limited information processing; rather, under certain circumstances, NFC may lead to a more complex decision making style (e.g., Kruglanski et al. 1991; Vermeir et al. 2002). Although most of the previous effects of NFC involved simplified cognitive processes such as the anchoring effect (Kruglanski and Freund 1983), increased overattribution bias (Webster 1993), mere exposure effects (Kruglanski et al. 1996) or the effects of priming (e.g., Ford and Kruglanski 1995), and reliance on stereotypes (Dijksterhuis et al. 1996), these processes were all observed in situations where prior knowledge was easily accessible. We believe that such conditions are sufficient for high NFC individuals to make a decision or form a judgment based on a limited information search. However, in situations where there is no clear rule to follow, high NFC subjects prolong the information search supposedly to reach a satisfactory level of certainty.

One important limitation to the generalizability of our findings stems from the fact that we found differences between the FW and DW rounds. The pattern of results for the FW rounds was consistent across two studies but not for the DW rounds, which may suggest that the effects of BIS and NFC are different with regard to situations that involve losses and ambiguity as it was the case in the DW round. The lack of effect of NFC in this condition could be a result of two competing tendencies—to reduce uncertainty and to reduce costs—both of which may play a role for highly uncertain individuals. In such a case we suspect that the differences between conditions would be stronger if the gains and losses were more meaningful to participants than in our study. However, the lack of expected NFC effects might also be due to the lower variance of dependent variables in the DW conditions because on average, participants in the DW conditions opened fewer boxes to decrease the costs of making a decision and spent less time on the task.

The results of our study also contribute to existing knowledge on the sources of NFC. The strong and positive relationship between BIS and NFC may suggest that limited information processing observed under high NFC, which on the surface seems to be simple and effortless, may instead result from higher emotional costs experienced by high BIS individuals in uncertain situations. Stress and cognitive effort resulting from sensitivity to uncertainty may be responsible for the typical effects of NFC on judgment formation and decision making, which involves a shorter and less extensive search. Simultaneous inclusion of measures of cognitive resources and motivational factors as predictors of NFC would help to clarify the origins of NFC in future studies.

One of the limitations of our study is that there were more female than male participants. Gender may be an important factor determining the relationships explored in our study as previous studies have demonstrated higher scores of BIS among women than among men (e.g., Carver and White 1994). The interesting question here is whether men and women satisfy their search for certainty in similar ways or whether they apply different decision making strategies to decrease the feeling of uncertainty. Therefore, it would be important to investigate this question in a more gender-balanced group. Our studies have also certain limitations resulting from the use of the Information Sampling Task. One limitation stems from the fact that the gains and costs experienced by participants were artificial and without real consequences. It could be expected that if the decisions made by participants brought real-life consequences such conditions could elicit stronger effects than the ones observed in laboratory conditions. On the other hand, the most popular measures of social value orientation are based on decomposed games, such as Triple Dominance Measure (Van Lange et al. 1997), and they use abstract point allocations. The research accumulated so far confirms the validity and accuracy of such measures for real-life allocation decisions. Finally, in our study participants received information about the accuracy of their decision, which could increase their confidence in a chosen strategy. Without such feedback, we would expect typical effects of anxiety and NFC to occur, such as escaping from a stressful situation by giving the first plausible answer.

In summary, we believe that our research, despite its limitations, offers the first attempt to integrate the basic personality variable represented by BIS and a specific epistemic motivation in a coherent model and to verify the simultaneous effect of those factors on decision-making style.

## Footnotes

1 The regression diagnostics statistics were much higher for this case than for other cases (Cook’s distance = 0.19, Studentized residual = −2.74, Standardized DFFIT = −0.79).
